# The Diagnostic Dilemma of Dieulafoy’s Lesion

**DOI:** 10.14740/gr671w

**Published:** 2015-07-22

**Authors:** Rafay Khan, Abdul Mahmad, Mark Gobrial, Francis Onwochei, Kunal Shah

**Affiliations:** a Internal Medicine Department, Raritan Bay Medical Center, 530 New Brunswick Avenue, Perth Amboy, NJ 08861, USA; b Internal Medicine Department, Maimonides Medical Center, 4802 10th Ave, Brooklyn, NY 11219, USA; c Internal Medicine Department, Jersey City Medical Center, 355 Grand St, Jersey City, NJ 07302, USA; d St. George’s University School of Medicine, University Centre, Grenada, West Indies

**Keywords:** Dieulafoy’s lesion, Hemorrhage, Gastrointestinal bleeding, Endoscopy, Diagnosis

## Abstract

Dieulafoy’s lesion (DL) is a relatively rare condition which carries a significantly high risk for mortality. A tortuous large arteriole in the wall of the stomach can result in significant gastrointestinal (GI) hemorrhage which can result in detrimental complications. Although it only accounts for about 1% of all GI bleeding, it has been considered to be one of the most underrecognized conditions. This train of thought may unfortunately be related to the difficulty in its diagnosis. After conducting a Medline search of the medical literature, with a focus on current PubMed articles, a thorough examination of updated diagnostic and treatment approaches was compared. Diagnostic techniques in the analysis and treatment of DLs continue to be limited to this day. Endoscopy remains as the main diagnostic and therapeutic tool; however, it continues to have its limitations. Other alternatives include but are not limited to angiography and surgical interventions which at times can be more successful. Diagnostic improvements and research for the detection of DL continue to advance; however, they remain limited in their capabilities. Further analysis and workup needs to be conducted in order to reduce hospital stay and improve survival.

## Introduction

Dieulafoy’s lesion (DL) is considered a significantly large submucosal artery which can erode the mucosa and result in bleeding via the digestive tract through a small mucosal defect [1, 2]. Paul Dieulafoy in 1897 was a professor of pathology at the Faculty of Medicine in Paris and was the first to describe this relatively rare condition. He presented a series of 10 patients with massive hematemesis due to a bleeding gastric vessel without any evidence of an ulceration in the first three lesions. With autopsy, a superficial ulceration was identified and consisted of a gaping arteriole which was found within the gastric submucosa. He concluded that the lesion was not a typical form of gastric ulcer and identified it as “exulceratio simplex” which later became known as Dieulafoy lesion [3, 4]. At the current time, endoscopy continues to be implemented as the main diagnostic approach in identifying not only DL but most gastrointestinal (GI) forms of bleeding. Furthermore, the advent of endoscopy has significantly impacted the treatment of DL, with forms such as laparoscopic banding, injections with epinephrine and adrenaline, and thermocoagulation; yet the success rate of these diagnostic and therapeutic approaches continues to be limited.

## Literature Search Method

Medical databases consisting of Medline and specifically revolving on publications found on PubMed were accessed and reviewed. Search criteria consisted of “Dieulafoy’s lesion”, “hemorrhage“, “bleeding”, and “GI” which allowed the retrieval of several updated and accessible works from the medical database. Types of articles mostly consisted of but were not limited to case reports, review articles, and research articles involving clinical data. A consensus was formulated incorporating the combination of these sources and was collaborated in order to give an updated review on this condition.

## Etiology and Epidemiology

In all cases of non-varicose bleeding in the upper GI tract, 6% consist of DL [5, 6]. While in all forms of GI hemorrhages, 1-2% are a result of DL [7, 8]. Although the lesion can occur at any age, the mean age demonstrated in the literature has been in the fifth decade of life and without any familial predisposition [7, 9]. It has also been found to be twice as more common in males than females [7]. Some associations have been documented, as with any form of GI bleeding are the use of anti-inflammatory medications, aspirin, and anti-platelet aggregation agents [7, 9-11].

**Figure 1:**
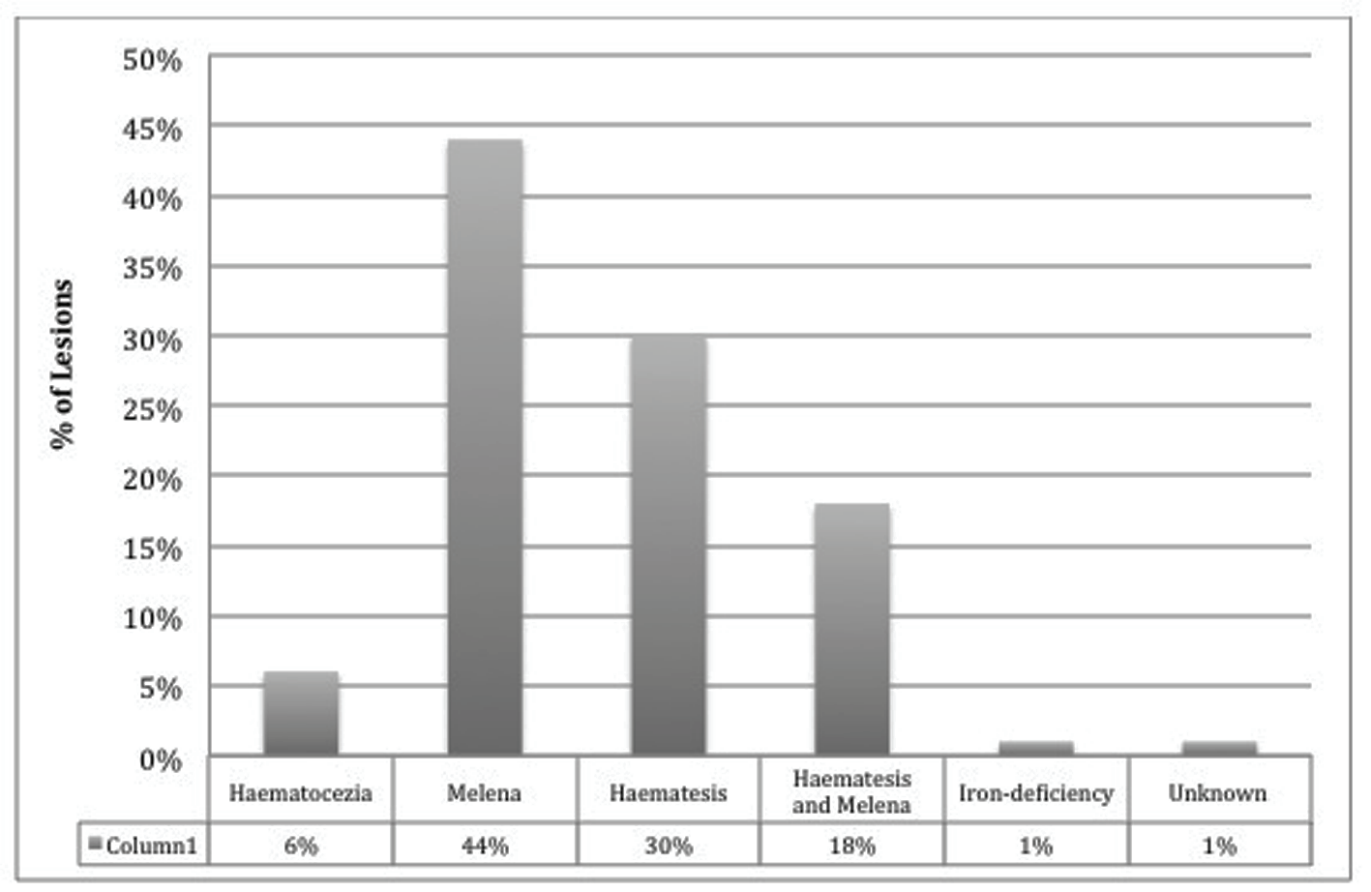
DL categorized by symptomatic presentation.

Majority of cases arise and present as a sudden onset of massive, recurrent, and painless hematemesis, although can also present as melena, hematochezia, and a drop in blood pressure. Forty-four percent of patients report melena, 30% hematemesis, while 18% have both hematemesis and melena, 6% only with hematochezia, and 1% present as iron-deficiency anemia [3, 12] (Fig. 1). The mean reported hemoglobin that patients may present with has been reported to be 8.4 and 9.2 g/dL on admission [3, 13], although this can be highly variable and fluctuate at a rapid rate.

## Pathology

It has been over a hundred years since these lesions were described; however, the pathogenesis and mechanisms resulting in tortuosity of these massive submucosal arteries continue to remain an enigma. There has been on-going debate in regards to its correlation with the underlying mucosa resulting in several detrimental complications. The actual etiology behind the mucosal rupture also remains undiscovered. It was once thought to be associated with an acquired aneurysm but further research has failed to prove a link to aneurysms, arteriosclerosis, elastic tissue dysfunction, or vasculitis [1, 7, 9, 10].

One theory has been described considering the idea that a mechanism resulting in pulsation within the large submucosal artery may apply pressure to the epithelium resulting in small erosion and rupture of the vessel towards the lumen [9, 12, 14]. However, it has been demonstrated that often times the lesions tend to be intermittent in nature. Another hypothesis has suggested that a thrombosis within the artery itself may result in continuous necrosis of the arterial walls, which can in turn lead to arterial rupture [1, 7, 9].

**Figure 2:**
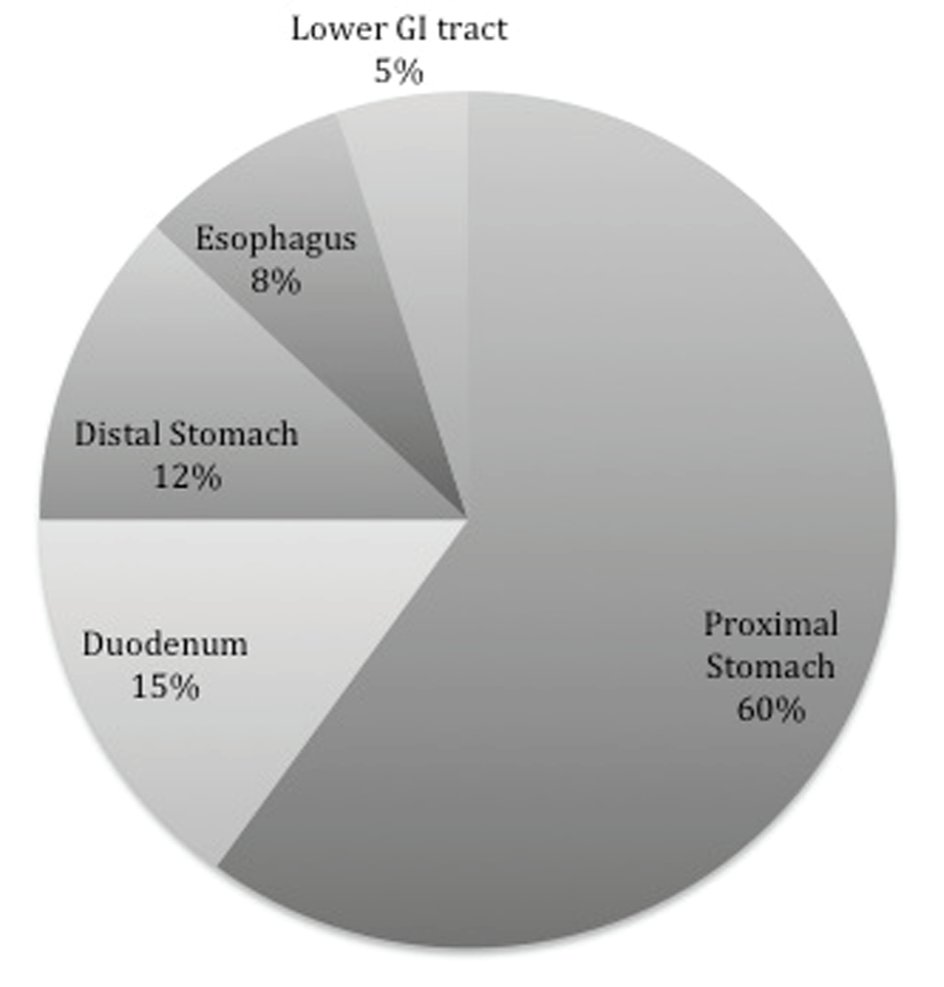
Location by percentage of Dieulafoy’s lesion within the GI system.

It has been further emphasized that as DL can present in several different locations within the GI system, there may be multiple mechanisms of action behind its formation depending on the location. The most common location reported has been the stomach, specifically the lesser curvature [15-17]. Of these 80-95% were found located within 6 cm of the gastroesophageal junction, which may be due to the direct correlation with the left gastric artery [15, 18, 19]. The remainder of the lesions consist of about a third of DL identified and are located mostly in the duodenum [20], followed by the colon [21], but have also been described in the esophagus, jejunum, and ileum [22], as well as the rectum [23] and even the anal canal [15, 24] (Fig. 2).

It remains unclear if these lesions are inherited or acquired but no genetic mutations have yet to be discovered. Although older ages of patients frequently found to have DL may lean toward an acquired phenomenon, the tendency for the vast majority of lesions to be found within 6 cm of the gastroesophageal junction questions whether an underlying congenital defect at that location is present or whether the location is more prone to acquired alterations. The pediatric literature however does demonstrate that the tortuous artery with variable length may be a congenital phenomenon, yet there still is a lack of sufficient support [25].

## Diagnosis

As DL frequently has been found to bleed intermittently and can present in various locations that may be inaccessible by endoscopy such as the jejunum or ileum, it may be difficult to diagnose. Several of these lesions may also be small and relatively inconspicuous despite repetitive endoscopic and other diagnostic measures. This forms a dilemma and can result in increased mortality in patients compared to other forms of GI bleeding which can be easily managed.

The most commonly used diagnostic test is an esophagogastroduodenoscopy (EGD), which would demonstrate a pigmented protuberance from a vessel stump that may be surrounded with minimal erosion and lack of ulceration [26]. This pigmented protuberance can have a variable color, is approximately 10 - 15 mm wide, 5 - 10 mm high, and only 50-60% may be actively bleeding with minimal spurting or oozing of blood from the GI mucosa [26-28]. Several patients may require repetitive endoscopies, as initial diagnostic measures may not reveal a source; however, patient hemoglobin may continue to drop. It may not be detected because an adherent clot may occlude it and sometimes the only way to directly ex- pose it is by washing away the clot with moderate endoscopic perfusion although this is not highly recommended [26].

Initial EGD has been found to be diagnostic in only about 70% of cases, as the lesions are relatively small and intermittently active. They may also be located between folds, covered by a clot, located underneath gastric contents, or hidden due to pools of massive bleeding [29]. Endoscopic criteria which have been at times utilized to define DL include the following [5, 9]: 1) active arterial spurting or micropulsatile streaming from a < 3 mm defect in the mucosa or surrounding normal mucosa; 2) protruding vessel visualization either with or without active bleeding within the small mucosal defect or through normal surrounding mucosa; and 3) fresh, densely adherent clot appearance with a minute point of attachment of mucosa. Although the final yield of diagnostic EGD is 70% for DL, the initial diagnostic findings are far less. Approximately 49% of lesions are identified during the initial EGD and 33% may require more than one EGD to correctly locate the active source of bleeding [9, 30].

As EGD has its limitations, push enteroscopy has also been utilized which may have a higher yield in identifying obscure locations with DL. Enteroscopy is often indicated after EGD and colonoscopy have failed to be diagnostic. It allows for viewing of the GI system about 150 cm beyond the pylorus and is able to identify distal duodenal or proximal jejunal lesions [26]. Wireless capsule endoscopy has also been implemented and although it has been considered minimally invasive, it lacks the benefit of therapeutic intervention [9, 31]. Capsule endoscopy is a way of investigating areas of GI tract which can be difficult to identify with EGD and even colonoscopy. With its use, there continues to be limitation as the camera may miss smaller lesions as it may not have the correct direction as it bypasses the lesion.

When EGD has failed to locate the source of bleeding, angiography has been implemented. On angiogram, the contrast extravasation into the eroded artery can be indicative of the lesion. It is more useful particularly in colorectal lesions where there may be poor bowel preparation, which can obscure colonoscopy results [12]. No angiographic pattern has been found to be specific for DL but may include findings such as visualization of a non-tapering, ectatic artery at the bleeding site [26]. Extravasation as stated may be the only finding at times. In one study, angiography was found to be diagnostic in 11 of 14 patients who had DL and who had undergone non-diagnostic endoscopic testing [26, 30].

Other diagnostic utilities that have been used include the additional use of endoscopic ultrasound [32]. Endosonographic features can demonstrate a large submucosal artery, which can be located at the lesser gastric curve near the gastroesophageal junction and is mostly used to confirm endoscopic hemostasis of a bleeding lesion by illustrating absent blood flow after therapy [26, 32]. This type of practice has not been used and is not often recommended due to its increased cost and lack of efficacy.

A final investigational measure which can be used when other diagnostic techniques have failed and the patient is clinically stable is a red cell scan with technetium-99m. The advantage of which it carries is that the threshold for detecting any extravasation into the gut is only 20% of that required by angiography [33]. However, again this testing modality is not often used because of the lack of data supporting its success rate compared to EGD.

## Treatment

Treatment options depend on a variety of factors including patient’s presentation, site of lesion, and diagnostic techniques utilized. Endoscopic methods remain the most often used treatment of choice in lesions which are easily accessible [17]. With the use of endoscopic measures, the treatment options are divided into three groups [12, 34, 35]: 1) thermal-electrocoagulation, heat probe coagulation and argon plasma coagulation; 2) regional injection-local epinephrine injection and sclerotherapy; and 3) mechanical- banding and hemoclip.

Prior to implementing these curative techniques, an attempt needs to be made to make the patient hemodynamically stable. It is vital to focus on volume resuscitation in order to prevent consequences of end-organ damage. Multiple large-bore, intravenous lines are inserted and volume resuscitation performed with crystalline solution, whether with normal saline or lactate ringers. Depending on the patient’s level of anemia, transfusion of packed red blood cells is often required and in most patients with DL it has been shown to require three or even more units [36]. DL requires hemostatic therapy, as often times re-bleeding can occur and the diagnosis, specifically the source of the bleeding, may not be initially discovered.

As stated, therapeutic endoscopy is the primary treatment and can achieve initial hemostasis in about 90% of lesions which are accessible and can decrease re-bleeding rates to less than 10% within the first 7 days [11, 26, 34, 37]. With the use of endoscopic hemostatic procedures, the various treatment techniques will be discussed.

Epinephrine injection and sclerotherapy is one of the treatments used to stop GI bleeding. Epinephrine with repeated injection can lead to cessation of bleeding. It is a relatively cheap treatment technique; however, it has been noted that alone in the management of DL it is not recommended due to the risk of re-bleeding [38]. Epinephrine around the lesion can be used to reduce excessive bleeding but should also be managed with sclerotherapy for those with DL. Sclerotherapy using ethanol or polidocanol has been shown to successfully control bleeding when used at four sites around the vessel and then into the vessel itself [9, 39].

Thermal coagulation, another useful measure, can be grouped depending on whether it is contact involving bipolar electrocoagulation and heater probe coagulation or non-contact, delivering high-frequency monopolar current through a conductive gas to the submucosa [9, 40]. Non-contact is considered advantageous as it can reduce the risk of perforation by decreasing the depth of tissue damage and due to the ease of its use [40]. Contact thermal methods, however, have been criticized because of inadequate coagulation of the lesion when covered by blood resulting in future episodes of re-bleeding [41].

Endoscopic band ligation and hemoclips are the most commonly used mechanical therapies. They have been shown to be more successful at times compared to injection treatment in the management of DL [9, 42]. Although the success rate may be higher, it only applies to those properly applied. It should be reserved for experienced endoscopists, especially when the angle is difficult, as incorrectly deployed hemoclips can prevent proper positioning of future clips [9].

Angiography with gel-foam embolization is a relatively rare form of management but can be an effective final resort if endoscopic treatment fails. Unfortunately, there may be a risk of ischemia to the area supplied by the artery which is embolized. Thus, when the bleeding lesion is supplied by multiple collateral vessels, this technique is not useful [12]. Surgery is the last option for patients with uncontrolled treatment but carries a higher mortality rate as often times these patients may already be hemodynamically unstable at the time surgery is considered.

## Conclusion

Although the locations and treatments used for DL are well documented, the etiology remains poorly understood and due to the intermittent nature of DL, diagnostic approaches have their limitations. In future studies, the developmental origin needs to be further investigated which may allow for better diagnostic techniques resulting in improved mortality rates and patient care. Significant research, data collaboration, and clinical trials need to be conducted to differentiate various endoscopic modalities and the best method to approach and manage such lesions. Endoscopic ultrasound and enteroscopy have yet to be used as often as EGD and are limited to certain facilities, with future use and implication it is possible to determine which method of diagnosis will lead to an early discovery and treatment thus preventing deteriorations in patients’ condition and decreasing the need for recurrent scoping. The risk of re-bleeding from DL has been reported to vary anywhere between 9% and up to 40% and has been found to by higher in endoscopic monotherapy compared with combined endoscopic therapies [8, 10, 12]. Although advances in endoscopy have in fact improved the detection rate from 80% [10, 12] to 86% [12, 43], there remains room for improvement. GI endoscopy has proven to be an effective diagnostic and therapeutic tool but the obscure nature of DL reveals that there is a significant amount of underlying investigation that needs to be conducted.

## References

[1] Juler GL, Labitzke HG, Lamb R, Allen R (1984). The pathogenesis of Dieulafoy's gastric erosion. Am J Gastroenterol.

[2] Tejero Cebrian E, Garcia Aguilar J, Arias Perez J, Alia Benitez J, Jorge Sanchez E, Duran Sacristan H (1987). Enfermedad de Dieulafoy: unacausarara de hemorragia digestive alta. Rev Esp Enferm Dig.

[3] Senger Jenna-Lynn, Kanthan Rani (2012). The Evolution of Dieulafoy's Lesion Since 1897: Then and Now—A Journey through the Lens of a Pediatric Lesion with Literature Review. Gastroenterology Research and Practice.

[4] Dieulafoy G, Dieulafoy G (1898). Exulceratio simplex: lecons 1-3. Clinique Medicale de l'HotelDieu de Paris.

[5] Stark ME, Gostout CJ, Balm RK (1992). Clinical features and endoscopic management of Dieulafoy's disease. Gastrointest Endosc.

[6] Strong RW (1984). Dieulafoy's disease--a distinct clinical entity. Aust N Z J Surg.

[7] Chaer RA, Helton WS (2003). Dieulafoy's disease. J Am Coll Surg.

[8] Marangoni G, Cresswell AB, Faraj W, Shaikh H, Bowles MJ (2009). An uncommon cause of life-threatening gastrointestinal bleeding: 2 synchronous Dieulafoy lesions. J Pediatr Surg.

[9] Jeon HK, Kim GH (2015). Endoscopic Management of Dieulafoy's Lesion. Clin Endosc.

[10] Lee YT, Walmsley RS, Leong RW, Sung JJ (2003). Dieulafoy's lesion. Gastrointest Endosc.

[11] Baettig B, Haecki W, Lammer F, Jost R (1993). Dieulafoy's disease: endoscopic treatment and follow up. Gut.

[12] Baxter M, Aly EH (2010). Dieulafoy's lesion: current trends in diagnosis and management. Ann R Coll Surg Engl.

[13] Njeru M, Seifi A, Salam Z, Ognibene L (2009). Dieulafoy lesion: a rare cause of gastrointestinal bleeding. South Med J.

[14] Fockens P, Tytgat GN (1996). Dieulafoy's disease. Gastrointest Endosc Clin N Am.

[15] Jamanca-Poma Y, Velasco-Guardado A, Pinero-Perez C, Calderon-Begazo R, Umana-Mejia J, Geijo-Martinez F, Rodriguez-Perez A (2012). Prognostic factors for recurrence of gastrointestinal bleeding due to Dieulafoy's lesion. World J Gastroenterol.

[16] Lara LF, Sreenarasimhaiah J, Tang SJ, Afonso BB, Rockey DC (2010). Dieulafoy lesions of the GI tract: localization and therapeutic outcomes. Dig Dis Sci.

[17] Schmulewitz N, Baillie J (2001). Dieulafoy lesions: a review of 6 years of experience at a tertiary referral center. Am J Gastroenterol.

[18] al-Mishlab T, Amin AM, Ellul JP (1999). Dieulafoy's lesion: an obscure cause of GI bleeding. J R Coll Surg Edinb.

[19] Anireddy D, Timberlake G, Seibert D (1993). Dieulafoy's lesion of the esophagus. Gastrointest Endosc.

[20] Pollack R, Lipsky H, Goldberg RI (1993). Duodenal Dieulafoy's lesion. Gastrointest Endosc.

[21] Katsinelos P, Pilpilidis I, Paroutoglou G, Galanis I, Tsolkas P, Fotiadis G, Kapelidis P (2004). Dieulafoy-like lesion of the colon presenting with massive lower gastrointestinal bleeding. Surg Endosc.

[22] Dulic-Lakovic E, Dulic M, Hubner D, Fuchssteiner H, Pachofszky T, Stadler B, Maieron A (2011). Bleeding Dieulafoy lesions of the small bowel: a systematic study on the epidemiology and efficacy of enteroscopic treatment. Gastrointest Endosc.

[23] Yoshikumi Y, Mashima H, Suzuki J, Yamaji Y, Okamoto M, Ogura K, Kawabe T (2006). A case of rectal Dieulafoy's ulcer and successful endoscopic band ligation. Can J Gastroenterol.

[24] Firat O, Karakose Y, Caliskan C, Makay O, Ozutemiz O, Korkut MA (2007). Dieulafoy's lesion of the anal canal: report of a case. Turk J Gastroenterol.

[25] Linhares MM, Filho BH, Schraibman V, Goitia-Duran MB, Grande JC, Sato NY, Lourenco LG (2006). Dieulafoy lesion: endoscopic and surgical management. Surg Laparosc Endosc Percutan Tech.

[26] Nojkov B, Cappell MS (2015). Gastrointestinal bleeding from Dieulafoy's lesion: Clinical presentation, endoscopic findings, and endoscopic therapy. World J Gastrointest Endosc.

[27] Lopez-Arce G, Zepeda-Gomez S, Chavez-Tapia NC, Garcia-Osogobio S, Franco-Guzman AM, Ramirez-Luna MA, Tellez-Avila FI (2008). Upper gastrointestinal dieulafoy's lesions and endoscopie treatment: first report from a mexican centre. Therap Adv Gastroenterol.

[28] Lim W, Kim TO, Park SB, Rhee HR, Park JH, Bae JH, Jung HR (2009). Endoscopic treatment of dieulafoy lesions and risk factors for rebleeding. Korean J Intern Med.

[29] Chung YF, Wong WK, Soo KC (2000). Diagnostic failures in endoscopy for acute upper gastrointestinal haemorrhage. Br J Surg.

[30] Reilly HF, al-Kawas FH (1991). Dieulafoy's lesion. Diagnosis and management. Dig Dis Sci.

[31] Lai LH (2008). Obscure GI bleeding: is capsule endoscopy sufficient?. Gastrointest Endosc.

[32] Jaspersen D (1993). Dieulafoy's disease controlled by Doppler ultrasound endoscopic treatment. Gut.

[33] Jensen DM (2001). Endoscopic diagnosis and treatment of severe hematochezia. Tech Gastrointest Endosc.

[34] Chung IK, Kim EJ, Lee MS, Kim HS, Park SH, Lee MH, Kim SJ (2000). Bleeding Dieulafoy's lesions and the choice of endoscopic method: comparing the hemostatic efficacy of mechanical and injection methods. Gastrointest Endosc.

[35] Pathan NF, El-Fanek H (2006). A 70-year-old man with episodes of upper gastrointestinal bleeding. Dieulafoy lesion/malformation. Arch Pathol Lab Med.

[36] Cappell MS, Yamad T, Alpers D, Kalloo (2009). Gastrointestinal vascular malformations or neoplasms: Arterial, venous, arteriovenous and capillary. Textbook of Gastroenterology.

[37] Kasapidis P, Georgopoulos P, Delis V, Balatsos V, Konstantinidis A, Skandalis N (2002). Endoscopic management and long-term follow-up of Dieulafoy's lesions in the upper GI tract. Gastrointest Endosc.

[38] Pointner R, Schwab G, Konigsrainer A, Dietze O (1988). Endoscopic treatment of Dieulafoy's disease. Gastroenterology.

[39] Skok P (1998). Endoscopic hemostasis in exulceratio simplex-Dieulafoy's disease hemorrhage: a review of 25 cases. Endoscopy.

[40] Canard JM, Vedrenne B (2001). Clinical application of argon plasma coagulation in gastrointestinal endoscopy: has the time come to replace the laser?. Endoscopy.

[41] Lin HJ, Tsai YT, Lee SD, Lai KH, Lee FY, Lin CY, Lee CH (1988). A prospectively randomized trial of heat probe thermocoagulation versus pure alcohol injection in nonvariceal peptic ulcer hemorrhage. Am J Gastroenterol.

[42] Park CH, Sohn YH, Lee WS, Joo YE, Choi SK, Rew JS, Kim SJ (2003). The usefulness of endoscopic hemoclipping for bleeding Dieulafoy lesions. Endoscopy.

[43] Alshumrani G, Almuaikeel M (2006). Angiographic findings and endovascular embolization in Dieulafoy disease: a case report and literature review. Diagn Interv Radiol.

